# Rapid Enkephalin Delivery Using Exosomes to Promote Neurons Recovery in Ischemic Stroke by Inhibiting Neuronal p53/Caspase-3

**DOI:** 10.1155/2019/4273290

**Published:** 2019-03-04

**Authors:** Yang Liu, Naisheng Fu, Jiangli Su, Xiumei Wang, Xiaohua Li

**Affiliations:** ^1^The Department of Rehabilitation, Liaocheng People's Hospital, Liaocheng, Shandong 252000, China; ^2^Department of Internal Neurology, Liaocheng People's Hospital, Liaocheng, Shandong 252000, China; ^3^Department of Dermatology, Liaocheng People's Hospital, Liaocheng, Shandong 252000, China

## Abstract

No pharmacological treatment is currently available to protect brain from neuronal damage after ischemic stroke. Recent studies found that enkephalin may play an important role in neuron regeneration. We assembled a homogeneous size vesicle constituted by transferrin, exosomes, and enkephalin. Immunofluorescence assay showed that transferrin was combined with the exosomes and enkephalin was packaged into the vesicle; thus this complex was called tar-exo-enkephalin.* In vitro* studies were performed using rat primary hippocampal neurons and the results showed that enkephalin decreased p53 and caspase-3 levels to 47.6% and 67.2%, respectively, compared to neurons treated with glutamate, thus inhibiting neuron apoptosis caused by glutamate. An* in vivo* experiment in rats was also carried out using a transient middle cerebral artery occlusion (tMCAO)/reperfusion model and tar-exo-enkephalin treatment was performed after tMCAO. The results showed that tar-exo-enkephalin crossed the blood brain barrier (BBB) and decreased the levels of LDH, p53, caspase-3, and NO by 41.9, 52.6, 45.5, and 57.9% compared to the tMCAO rats, respectively. In addition, tar-exo-enkephalin improved brain neuron density and neurological score after tMCAO. These findings suggest that the use of exogenous enkephalin might promote neurological recovery after stroke.

## 1. Introduction

Ischemic stroke is one of the leading causes of morbidity worldwide, compromising human health due to its high incidence, death rate, and high recrudescence rate [1–3]. People who suffered brain damage because of stroke can develop new neural connections and regain some of their old skills with a proper rehabilitation, but at present, no pharmacological treatment is available to protect brain tissue from neuronal damage triggered by ischemia and reperfusion due to the blood brain barrier (BBB) inhibiting or limiting the access of medications to the brain [4, 5]. Increasing the dose of medicines may increase availability to the target tissue, although also increasing the risk of side effect [6, 7]. Therefore, targeted delivery of adequate neuron-protective medicines to the cerebral ischemia zone has become a current research focus of stroke's therapy. Exosomes are extracellular vesicles produced by all cells and naturally found in the blood. Exosomes derived from mesenchymal cells can avoid being phagocytosed by monocytes and macrophages because of CD47 and CD63 on their surface [8–10]. However, their use in targeted therapy is still under discussion.

Human iron metabolism is essential to maintain homeostasis and health. Transferrin receptor is involved in the regulation of iron metabolism. It is expressed at low level in quiescent cells, but it increases during active proliferation. After stoke, the transferrin receptor on the BBB is highly expressed, while the expression of this receptor in other cells is low [11]. Therefore, it is potentially possible to transfer exogenous drugs into the brain using the transferrin receptor on the BBB to cross it.

Many traditional medicines used in the treatment of stroke have a central nerve-depressant effect. Enkephalin is a neurotransmitter that can reduce pain, induce euphoria, and produce analgesia or anti-nociception via binding to opioid receptors like *δ* receptors [12]. Previous studies suggested that the activation of *δ* receptors opioid results in a significant action against oxidative stress in the central nervous system, promoting neurons' survival [13]. Thus, we hypothesized that exogenous enkephalin might increase its concentrations in the brain, in turn resulting in improved outcomes after stroke.

In this study, we hypothesized that enkephalin treatment might be a promising strategy to promote neuron regeneration through its ability to cross the BBB. To demonstrate our hypothesis, transferrin expression vectors were transfected into mesenchymal cells, exosomes derived from mesenchymal cells were extracted, and the so-called “tar-exo-enkephalin” vesicles were obtained and loaded with enkephalin. A series of experiments were performed to verify that these vesicles crossed the BBB after stroke and the consequent neuroprotection of enkephalin.

## 2. Materials and Methods

### 2.1. Cell Culture

Transgenic Bone Mesenchymal Stem Cells (BMSC, RASMX-01001, Cyagen Biosciences, Beijing) were cultured in low glucose DMEM (DMEM-LG), containing 10% fetal bovine serum (FBS, 10099141; Gibco; Thermo Fisher Scientific, Inc.), and incubated at 37°C. The transferrin expressing vector was transferred into the BMSC genome before culture.

Rat primary hippocampal neurons (RC002, Fenghui biology, Changsha, China) were cultured in DMEM/F12 containing EGF (20 ng/ml, Invitrogen, Carlsbad, CA, USA), bFGF (20 ng/ml, Invitrogen, Carlsbad, CA, USA), B-27 (2%, Invitrogen, Carlsbad, CA, USA), and antibiotic (1%, Invitrogen, Carlsbad, CA, USA) and were incubated at 37°C.

For* in vitro* experiments, rat hippocampal neurons were divided into three groups and stimulated with either 100 *μ*g/ml glutamate (dissolved in PBS, GLU, Sigma-Aldrich; Merck KGaA, Darmstadt, Germany) (n=8) or 100 *μ*g/ml GLU and 100 *μ*g/ml enkephalin (dissolved in PBS, n=8) for 48 h. The control group was treated with an equal volume of PBS (n=8).

### 2.2. Exosome Isolation

Exosomes were isolated as previously described after BMSC were cultured with DMEM-LG at 37°C for 2 days. The cell culture supernatant was subjected to ultra-high speed centrifugation (2000×g for 10 min, 10, 000×g for 30 min, 100, 000×g for 60 min, and 100, 000×g for 60 min) to obtain exosomes that BMSC secreted [14]. Then, electroporation was performed at 500V and 150 *μ*F to load exosomes with enkephalin using electroporation cuvettes (BTX ECM2001, Harvard Apparatus, America).

For exosomes cryo-electron microscopy (cryo-EM) analysis, exosomes were rapidly frozen in liquid nitrogen. Next, a R2x2 Quantifoil electron microscope (Micro Tools GmbH, Jena, Germany) was used to collect morphological information (20,000x) [15].

Nanoparticle tracking analysis (Nano series, Malvern, UK) was used to determine exosomes size. Results were performed in 20 runs per each construct after exosomes (15 *μ*l) were diluted in water [16, 17].

### 2.3. Western Blot

Total proteins from BMSC, hippocampal neurons, and exosomes were extracted by RIPA lysis buffer and separated by 6–12% sodium dodecyl sulfate-polyacrylamide gel electrophoresis (SDS-PAGE). Then, proteins were transferred onto a polyvinylidene difluoride membrane. As regards BMSC and exosomes analysis, they were incubated with the following primary antibodies: anti-HSP70 (Abcam), anti-TSG101 (Abcam), and anti-CD63 (Abcam) at 4°C for 12 h. As regards hippocampal neuron analysis, they were incubated with the following primary antibodies: anti-p53 (Abcam) and anti-caspase-3 (Abcam) at 4°C for 12. [18]. The anti-GAPDH antibody (Abcam) was used as loading control.

### 2.4. Real-Time PCR

GoldScript one-step RT-PCR Kit (Life Technologies, Carlsbad, USA) was used to measure the relative p53 and caspase-3 gene expression in rat primary hippocampal neuron of each group. Procedures, including the ones related to RT-PCR conditions, were strictly carried out by following the kit instructions. GAPDH was used as the housekeeping gene. Data were obtained by a Real-Time PCR Instrument (QuantStudio™ 3&5, ThermoFisher, Waltham, USA). The relative gene expression was calculated by the 2-ΔΔCT method. [19].

Primers were obtained from Genebio (Shanghai, China) and the primer sequences of p53, caspase-3, and GAPDH were as follows:

*p53*

  5′-GAGCGAATCACGAGGGAC-3′
  5′-GCACAAACACGGACAGGA-3′;

*Caspase-3*

  5′-TGGAACAAATGGACCTGTTGACC-3′
  5′-AGGACTCAAATTCTGTTGCCACC-3′;

*GAPDH*

  5′-GCACCGTCAAGGCTGAGAAC-3′
  5′-TGGTGAAGACGCCAGTGGA-3′.

### 2.5. Flow Cytometry

After cells of each group were cultured for 48 h, hippocampal neurons were harvested and resuspended in PBS. An Annexin V-PI Apoptosis Detection Kit (BD Biosciences, Franklin, New Jersey, USA) was used to identify apoptotic hippocampal neurons. Procedures were strictly performed by following the kit instructions and a laser flow cytometer (CyFlow® Cube, Partec, Germany) was used to evaluate cell apoptosis.

### 2.6. Animal tMCAO Model

A total of 100 Sprague Dawley rats (age, 8-12 weeks; weight, 220-240 g) were selected from the Experimental Animal Center of Liaocheng People's Hospital 5 days before the operation. All experiments that involved animals in this study were approved by medical ethics committee of Liaocheng People's Hospital. Animals were housed at 22-25°C with a humidity of 65±5%, 0.03% CO2, and 12 h light/dark cycle, with free access to water and food. A transient middle cerebral artery occlusion (tMCAO) model was established in rats as previously described [20–22]. In brief, the common, internal, and external carotid arteries were exposed by blunt dissection after 2.5% sodium pentobarbital injection (36 mg/kg; Sigma-Aldrich; Merck KGaA) into the rats' abdominal cavity. Rats were kept at 37°C. A 5-cm long nylon filament (diameter, 0.24-0.28 mm; Biospes Co., Ltd., Chongqing, China) was then inserted into the cut of the internal carotid artery for 2 h. Finally, the rats in the experimental group were treated with exosomes for 12 h and Tar-exosomes for 4 h or 12 h by injection into vein, while the rats without any surgery (sham group) were treated with 500 *μ*l exosomes (1x105/ml) for 12 h and 500 *μ*l Tar-exosomes (1x105/ml) for 12 h. Another experiment was performed, in which rats were treated with enkephalin-tar-exo or enkephalin-exo, while the rats without any surgery and rats that were subjected to tMCAO were treated only with saline (injected into vein, 100 mg/kg; Sigma-Aldrich; Merck KGaA). Ten rats were used for each group.

At 1 week and 3 weeks after treatments, neurological examination was carried out as previously described [23]. And then, after euthanasia by 2.5% sodium pentobarbital, brains were removed and cryostat brain sections (20 *μ*m) were cut using a cryomicrotome (HM525 NX; Thermo Fisher Scientific, Inc., Waltham, MA, USA). Cresyl violet (176022, J&K Chemical, Beijing, China) was used to stain sections following a previously reported method [24].

### 2.7. Immunofluorescence

Exo and tar-exos extracted by BMSC were incubated with the monoclonal antibody Rhoda-rats-anti-transferrin (1: 500, Abcam) for 12 h at 4°C, followed by rhodamine-conjugated rabbit anti-rat IgG secondary antibody for 90 min at room temperature. After high speed centrifugation (4°C, 1000 g, 1 h), exo and tar-exo were imaged using a fluorescence microscope at 590 nm. On the other hand, tar-exo were cocultured with hippocampal neurons at 37°C and fluorescence microscope was also used to image at 0.5 h, 1 h, and 2 h.

Cryostat brain sections were incubated with rat-anti-Iba1 (Abcam) for 12 h at 4°C and stained with a FITC-conjugated rabbit anti-rat IgG secondary antibody for 90 min at room temperature.

In addition, brain tissue sections were incubated for 12 h at 4°C with a mouse-anti-NeuN antibody (1: 600, Chemicon, Temecula, CA, USA). After washing with PBS, sections were incubated with a secondary antibody, FITC-conjugated rabbit anti-mouse immunoglobulin G (IgG, 1:600, ThermoFisher, Waltham, USA). Neurons were imaged using a fluorescence microscope at 525 nm.

### 2.8. ELISA

Rats' cerebrospinal fluid (CSF) was collected at 3 days and 7 days after tMCAO according to a reported method [25]. In addition, after electroporation, tar-exo and tar-exo-enkephalin were collected after ultrasonic wave breaking. Then, lactate dehydrogenase (LDH), p53, and caspase-3 protein expression in CSF and enkephalin in tar-exo-enkephalin were measured using ELISA kits (yuanmu Co. Ltd. Wuhan, China; Bos-ter Bioengineering Co. Ltd. Wuhan, China). Absorbance was read by a microplate reader (Synergy HT, Biotech, Vermont, USA).

### 2.9. Nitric Oxide Detection

Total NO levels in the CSF of each group were measured by the Griess reagent kit (Invitrogen) [26]. Absorbance was read by a microplate reader at a wavelength of 540 nm.

### 2.10. Behavioral Assessments

Neurological examination was carried out at 1 and 3 weeks after tMCAO according to a reported method [23]. The first method is that Sprague Dawley rats were held gently by tail, suspended at about 60 cm above the bench, and monitored for forelimb flexion. The neurological examination was evaluated using scores from 0 to 3, and the severity of brain damage was considered as positively correlated with the neurological examination score: score 0: absence of a neurological deficit; score 1: failure to extend right paw fully; score 2: circling to the right; score 3: falling to the right; score 4: no spontaneous walking and depressed level of consciousness. The inclined board test was used to assess balance and coordination. The rats were placed on an inclined board (50x30cm), and the maximum inclination angles of the board when the rats could maintain their posture were recorded. After repeating the test five times, the average holding angle was calculated.

### 2.11. Statistical Analysis

GraphPad Prism statistical software (La Jolla, CA, USA) was used to perform statistical analysis. Results are expressed as mean ± standard deviation. Statistically significant difference was calculated by one-way analysis of variance (ANOVA) followed by Student-Newman-Keuls post hoc test. Data were considered statistically significant when* P*<0.05.

## 3. Results

### 3.1. Tar-Exo Acquisition and Enkephalin Incorporation in Tar-Exo

**Figure 1:**
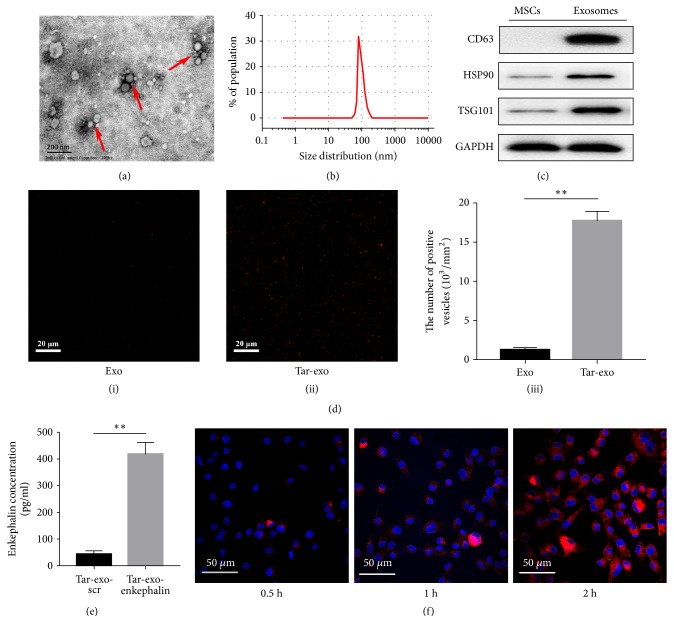
Tar-exo-enkephalin synthesis, formulation, and physicochemical features. (a) Cryo-EM analysis showed the morphology of tar-exo-enkephalin. (b) Nanoparticle tracking analysis showed the size of tar-exo-enkephalin. (c) Western blot of CD63, HSP70, TSG101, and GAPDH in exosomes and MSC cells. (d) Immunofluorescence assay confirming the binding of transferrin to the tar-exo membrane. (e) The level of enkephalin quantified by ELISA into tar-exo after electroporation. (f) Changes in exosomes number of neuron (blue) after treatment with tar-exo (red) obtained by immunofluorescence. Scale bar, 20 *μ*m (n=8 per group). Results are expressed as mean ± SD, *∗∗* and ##*P*< 0.01.

Transferrin expression vectors were transfected into BMSC. After tar-exo extraction, electroporation was performed using cuvettes, to load exosomes with enkephalin. Cryo-EM and nanoparticle tracking analysis results showed that tar-exo diameter ranged from 52.7 nm to 192.6 nm, with an average diameter of 127.4 nm (Figures 1(a) and 1(b)).

Western bolt results showed that the levels of the exosome markers CD63, HSP70, and TSG101 were higher in exosomes compared to BMSC (Figure 1(c)).

Since the protocol and treatment used did not damage the exosomes, the content of transferrin was evaluated in tar-exo and exosomes by immunofluorescence using an antibody directed against transferrin. The number of exosomes and tar-exo extracted was 1.78 × 10^3^/mm^2^ and 17.36 × 10^3^/mm^2^, respectively (Figure 1(d)). After ultrasonic wave breaking, the enkephalin levels of tar-exo-scr and tar-exo-enkephalin were tested by ELISA; they were 47.8 pg/ml and 428.1pg/ml (Figure 1(e)). Coculture of hippocampal neurons results showed that the number of tar-exo located into the neurons was increasing with time (Figure 1(f)).

### 3.2. Effect of Enkephalin on Hippocampal Neuron Apoptosis Induced by GLU

**Figure 2:**
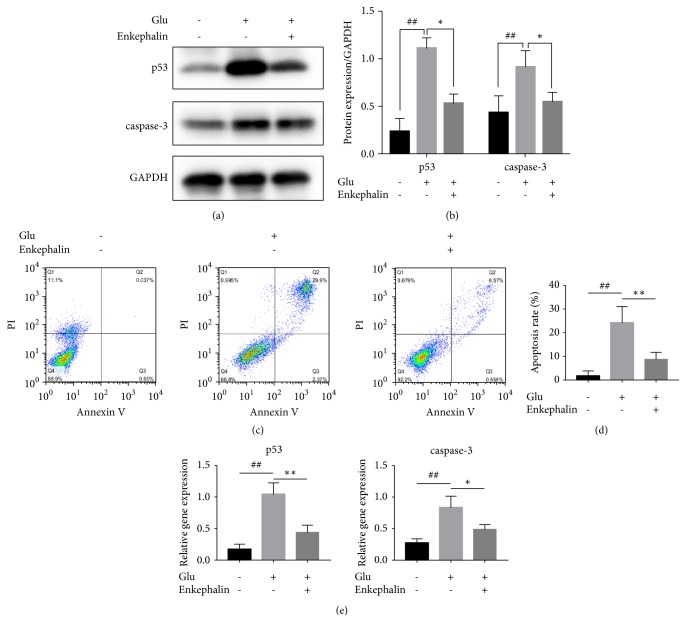
Effect of enkephalin on hippocampal neuron apoptosis induced by GLU. (a) Western blot of p53 and caspase-3 in hippocampal neurons. (b) p53 and caspase-3 gene expression detected by RT-PCR. (c) Annexin V-PI staining of hippocampal neurons and apoptosis percentage measured by flow cytometry (*n*=8 per group). Results are expressed as mean ± SD, *∗P*< 0.05, *∗∗* and ^##^*P*< 0.01.

Western blot results showed that p53/caspase-3 expression in rat primary hippocampal neurons was aberrant in GLU group compared to control group, while the expression of these proteins was back to normal after treatment with enkephalin after GLU (Figure 2(a)).

p53 and caspase-3 relative gene expression was 0.19 mg/ml and 0.26 mg/ml in untreated neurons measured by RT-PCR. Following stimulation with GLU, their levels increased by 497.8% and 205.2%, respectively (both* P*<0.01). However, their levels decreased to 47.6% and 67.2% as compared with GLU alone group when hippocampal neurons were treated with enkephalin after GLU (both* P*<0.01) (Figure 2(b)).

In addition, the apoptosis of hippocampal neurons was detected by flow cytometry. Apoptotic cells percentage was low in the control group (2.48%). After treatment with GLU, their percentage increased to 25.9% (*P*<0.01). However, the effect of GLU on apoptosis of hippocampal neurons was inhibited by enkephalin and the apoptotic percentage decreased to 9.7% (*P*<0.01) (Figure 2(c)).

### 3.3. Tar-Exo Transfer to the Ischemic Zone in tMCAO

**Figure 3:**
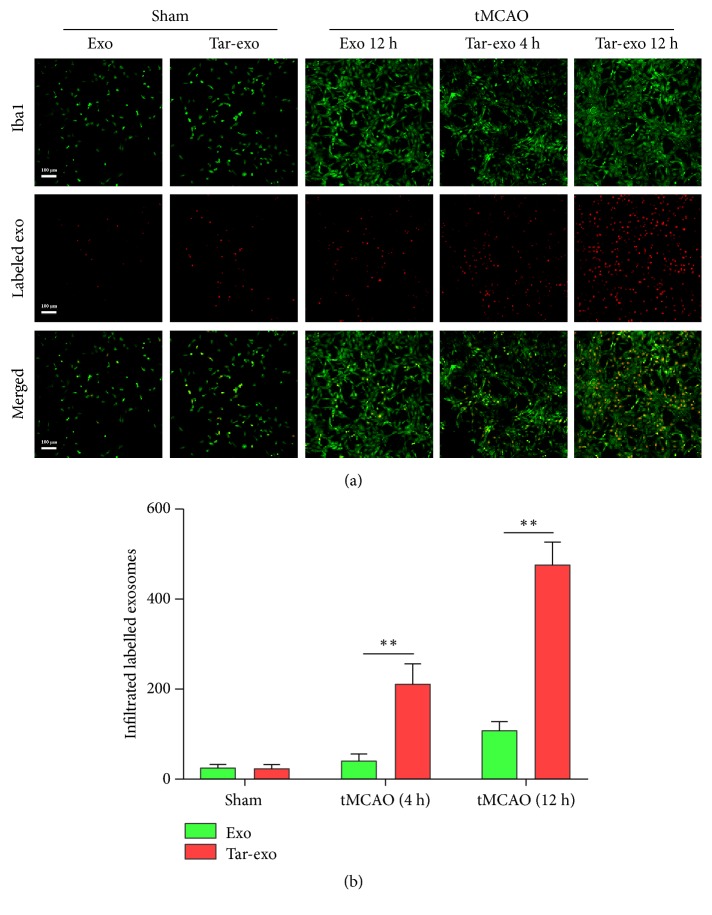
Tar-exo transfer to the ischemic zone in tMCAO. Rats were treated with exosomes or tar-exo at 2 h after tMCAO. Rats' brain sections were stained at 4 h and 12 h after vesicles treatment. Exosomes and tar-exo were stained with rhodamine (red) prior to injection, whereas Iba1 (green) was used to stain microglia. (a), (b) Representative images and quantification showing vesicles transfer and infiltration. Scale bar, 50 *μ*m (*n*=10 per group). Results are expressed as mean ± SD, *∗∗P*< 0.01.

In this study, transferrin was used as a target agent for the transfer of tar-exo to the ischemic zone. Exosomes and tar-exo were labeled with anti-TSG10. Immunofluorescence assay results showed that the total number of exosomes and tar-exo in the brain of the control group was 23.6/mm^2^ and 24.9/mm^2^, respectively, in the control group. However, at 4 h after tMCAO, the number of tar-exo that infiltrated into the brain increased to 223.5/mm^2^, while the exosomes number increased to 31.7/mm^2^. In addition, the number of tar-exo at 12 h after tMCAO further increased to 492.5/mm^2^, while the number of exosomes in the brain was 52.5/mm^2^. Tar-exo migration ability was higher than the one of exosomes. Our results also showed that many tar-exo were anti-Iba1 positive (Figures 3(a) and 3(b)).

### 3.4. Effect of Tar-Exo-Enkephalin on Neurons Injury Induced by tMCAO

**Figure 4:**
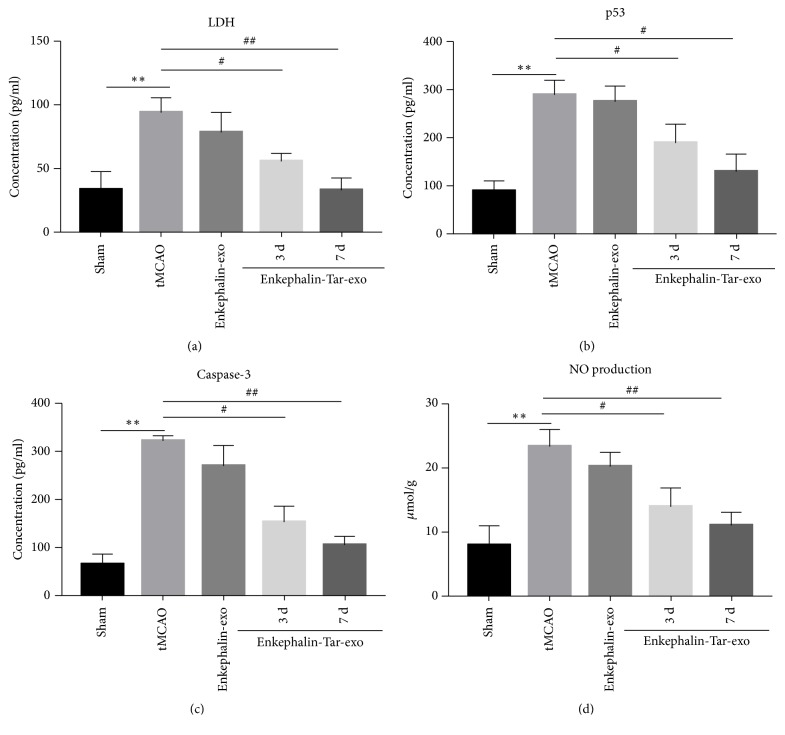
Effect of tar-exo-enkephalin on neurons injury induced by tMCAO. Rats were treated with enkephalin-exo or enkephalin-tar-exo at 2 h after tMCAO. CSF was collected at 3 and 7 days after vesicles treatment. ELISA and Griess reagent kit were used to measure LDH, p53, caspase-3, and NO levels in CSF. Results are expressed as mean ± SD, ^#^*P*< 0.05, *∗∗* and ^##^*P*< 0.01.

Following cerebral ischemia and reperfusion, many proinflammatory and cytotoxic factors, including IL-6, IL-1*β*, TNF-*α*, and NO, can cause cell injury and apoptosis. LDH, p53, caspase-3, and NO can be used as cell injury indexes. In the sham group, LDH, p53, caspase-3, and NO levels in the CSF were 38.6 pg/ml, 98.1 pg/ml, 66.4 pg/ml, and 8.9 *μ*mol/mg, respectively. After tMCAO those levels in the CSF were increased to 97.7 pg/ml, 296.1 pg/ml, 323.5 pg/ml, and 23.7 *μ*mol/mg, respectively (all* P*<0.01). Cell injury was significantly enhanced at 7 days. After treatment with enkephalin-exo, cell injury decreased, since those levels decreased by 87.2, 98.3, 91.3 and 93.5% (all* P*<0.01), respectively, compared with those observed in tMCAO group. However, treatment with enkephalin-tar-exo for 3 days resulted in LDH, p53, caspase-3, and NO decrease by 68.3, 72.7, 52.1, and 73.9%, respectively (all* P*<0.01), compared with those observed in tMCAO group. Furthermore, cell injury decreased more remarkably after 7 days, since LDH, p53, caspase-3, and NO levels decreased by 41.9 (*P*<0.01), 52.6 (*P*<0.05), 45.5 (*P*<0.01), and 57.9% (*P*<0.01) as compared with those observed in tMCAO group (Figure 4).

### 3.5. Effect of Tar-Exo-Enkephalin on Neuron Regeneration in tMCAO

**Figure 5:**
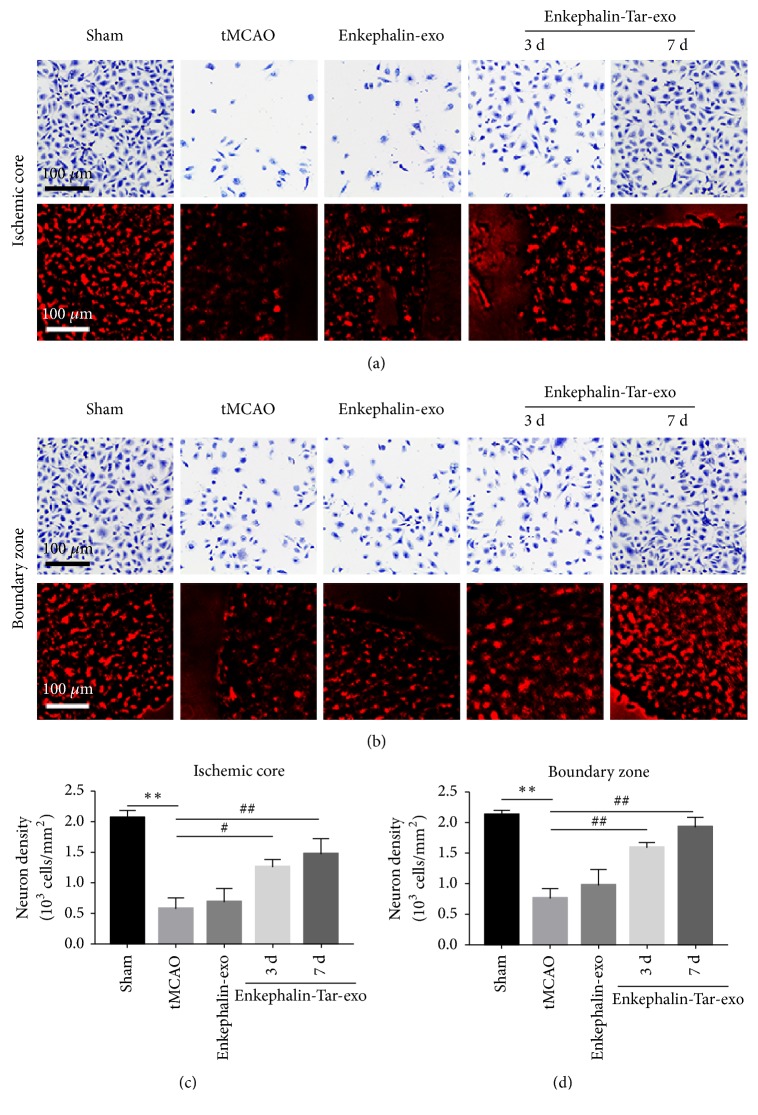
Effects of tar-exo-enkephalin on neuronal density in the post-ischemic brain. Rats were treated with enkephalin-exo or enkephalin-tar-exo at 2 h after tMCAO. Cresyl violet and NeuN (green) were used to stain neurons in the brain sections. At 1 week and 3 weeks after treatment with these vesicles, representative images and quantification showed the number and density of neuron in the ischemic core (a, c) and boundary zone (b, d). Scale bar, 50 *μ*m (*n*=10 per group). Results are expressed as mean ± SD, ^#^*P*< 0.05, *∗∗* and ^##^*P*< 0.01.

Neuronal apoptosis may be one of the most serious causes of neurological symptoms after stroke. Immunofluorescent staining against NeuN was performed on brain sections to determine neurons proliferation and processed with cresyl violet. In the sham group, neurons density in ischemic and boundary zones was 1.34 × 10^3^/mm^2^ and 1.19 × 10^3^/mm^2^, respectively. However, after tMCAO the cell density in both zones was decreased to 71.5% and 53.6%, respectively, compared with sham group. It suggested that the neurons located in both zones showed a normal morphology, and a significant neuronal damage was found in the ischemic core and the boundary zone at 3 weeks after tMCAO. After treatment with enkephalin-exo, no significant change in neuronal density was observed compared with the tMCAO group, since cell density in both areas was approximately 0.47 × 10^3^/mm^2^ and 0.52 × 10^3^/mm^2^, respectively. However, at 1 week after enkephalin-tar-exo, neurons in the ischemic and boundary zone showed a tendency to proliferate, and the neuronal density in both areas was increased to 0.76 × 10^3^/mm^2^ and 0.83 × 10^3^/mm^2^, respectively. Furthermore, the neuronal density in both areas was increased to 0.99 × 10^3^/mm^2^ and 1.26 × 10^3^/mm^2^, respectively, at 3 weeks after enkephalin-tar-exo (Figure 5).

### 3.6. Effect of Tar-Exo-Enkephalin on Neuronal Disorders in tMCAO

**Figure 6:**
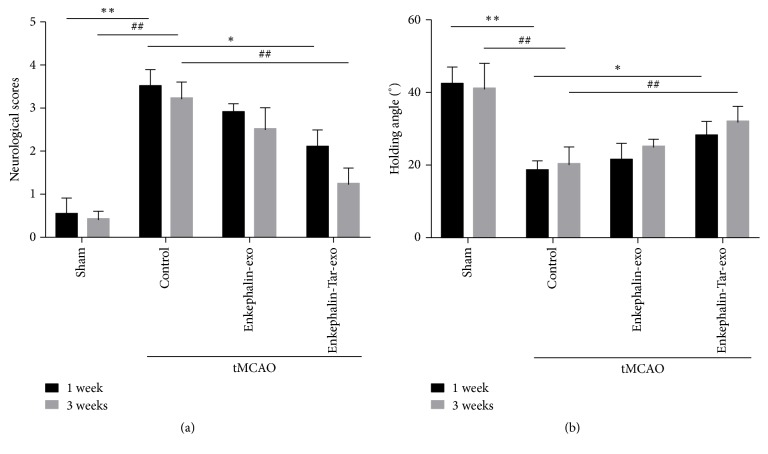
Effect of tar-exo-enkephalin on neuronal disorders in tMCAO. After injection with exo-enkephalin in tMCAO at 1 week and 3 weeks, the neurological scores both improve, as well as the increasing of holding angle in the inclined board test. Tar-exo-enkephalin also inhibited the ischemic injuries. n=10 per group. Results are expressed as mean ± SD, *∗* and #*P*< 0.05, *∗∗* and ##*P*< 0.01.

Neurological score and holding angle were tested in tMCAO at 1 and 3 weeks after surgery. The average score and angle of sham group were 0.43/41.3° and 0.39/42.5° in these time points. The neural function and motor function of SD rats in sham group were normal. After tMCAO, the neurological score was significantly improved compared with sham group, respectively (*P*<0.01). However, the results showed that the treatment with enkephalin-exo can reduce neurological score and increase holding angle (*P*<0.05). Furthermore, after being treated with enkephalin-tar-exo the average score and angle were changed to 2.21/28.9° and 1.34/36.5° at 1 week and 3 weeks. The neural function and motor function of SD rats attained improvement and protection (Figure 6).

## 4. Discussion

Neuronal death caused by ischemic stroke includes necrosis and apoptosis. Neuronal necrosis is irreversible, but apoptosis can be used as a potential target for the treatment of cerebral ischemia due to a large number of positive and negative regulatory signals [27–29]. In this study, specific vesicles were constructed using transferrin, enkephalin, and exosomes to specifically target neurons after crossing the BBB. The effects of these vesicles were explored* in vitro* and* in vivo* on the inhibition of neuronal apoptosis and promotion of neuronal regeneration.

Tar-exo-enkephalin using transferrin and enkephalin represents an innovative structure and approach in the treatment for stroke. Immunofluorescence showed that transferrin could be successfully bound to the surface of tar-exosomes and tar-exosomes could passively enter into neurons. ELISA results suggested that enkephalin was successfully transferred into the exosomes. On the other hand, the results obtained by the coculture of tar-exo and neurons for 2 h* in vitro* showed that tar-exo could passively enter into neurons.

In this study, flow cytometry was used to confirm that enkephalin could inhibit neuron apoptosis. In addition, WB and RT-PCR results suggested that enkephalin effect on apoptosis might be related to the inhibition of p53 and caspase-3.

A suitable drug delivery system is the ideal solution to improve the therapeutic effects. Because of the BBB, few exosomes were transferred into the brain in the sham group and the tMCAO model [30, 31]. However, many vesicles were transferred into the brain after treatment with tar-exo. The reason of this result might be due to the increased expression of the transferrin receptor on the BBB due to stroke. These results showed the target potential of transferrin on brain after ischemia and reperfusion, suggesting that also other brain diseases could be alleviated through the use of this targeting approach.

After confirming the targeting effect of tar-exo, our study also demonstrated why enkephalin targeting is needed. Indeed, neuron lesions degree and the levels of apoptotic related protein and cytotoxic factors were strongly decreased after treatment with enkephalin-tar-exo. The effect of enkephalin-tar-exo on the inhibition of brain injury was much stronger than the effect of enkephalin-exo. The main reason might be that enkephalin should be in a sufficient amount into the brain to exert its protective effect and enkephalin-exo vesicles could not guarantee the transport of the above mentioned sufficient amount into the brain due to the BBB because of the lack of target by these vesicles.

Neuronal cell death was increased after tMCAO, as shown by brain sections, and brain injury was not only limited to the ischemic zone. After treatment with enkephalin-tar-exo, neuronal regeneration was observed and continued for weeks. In addition, we also evaluated the neurological scores and the inclined board test, revealing that brain dysfunction due to ischemic-reperfusion injury was minimal after treatment with enkephalin-tar-exo. These results suggested that not only was the density of the neuron near normal, but also neuron functionality was recovered.

In conclusion, a novel carrier targeting the BBB was developed using enkephalin-loaded exosomes containing transferrin, called enkephalin-tar-exo, resulting in protecting brain from ischemia-reperfusion injury, providing a novel approach to reduce brain injury. In addition, side effects might be reduced because of the targeting ability of our vesicles and the natural substances representing the ingredients of our vesicle such as exosomes, enkephalin, and transferrin, although this aspect needs to be demonstrated. However, the ideal concentration of enkephalin-tar-exo and the specific underlying mechanisms in the reduction of brain apoptosis on nerve function regeneration still need further analysis, which will be the main focus of our future studies.

## Data Availability

The data used to support the findings of this study are available from the corresponding author upon request.
